# The Journal of Cognition after One Year: A Modern, Society-Backed, Fair Open-Access Option for Cognitive Psychology and Cognitive Neuroscience

**DOI:** 10.5334/joc.54

**Published:** 2019-01-24

**Authors:** Candice C. Morey

**Affiliations:** 1 School of Psychology, Cardiff University, UK

**Keywords:** Cognitive Psychology, Open access, ESCoP

In December 2016, the executive committee of the European Society for Cognitive Psychology (ESCoP) faced a momentous decision about whether to continue sponsoring and working on a subscription-based journal we did not fully control. We decided unanimously to abandon our journal, which was owned by its publisher, and instead establish an open-access journal focused on cognitive psychology and cognitive neuroscience. We saw an emerging need for an open-access journal with fair pricing, which embraced modern standards of transparency and openness about research methods while maintaining the quality and rigor expected from a traditional peer-reviewed journal. The Journal of Cognition, now ESCoP’s official journal, was born.

We have now completed our first volume, which includes 45 articles. Our first volume includes two special collections, one focusing on auditory distraction (https://www.journalofcognition.org/collections/special/the-confluence-of-sound-and-cognition/), organized by John Marsh and Rob Hughes, and another focusing on pupillometry (https://www.journalofcognition.org/collections/special/what-can-pupils-tell-us-about-cognition/), organized by Sebastian Mathôt. We featured a review article by Jan Theeuwes (2018) on attentional selection, along with critical, discussion-generating commentary (https://www.journalofcognition.org/collections/special/theoretical-review-with-commentaries-visual-selection/). We have published our first completed Registered Report (Lamy, Darnell, Levi, & Bublil, 2018), and others have been granted in-principle acceptance. Early indicators of our eventual debut impact factor (which will not officially appear until at least a year after we have published 2 volumes) look promising. At the end of this inaugural year, our content has been viewed or downloaded nearly 40,000 times. Our papers have already been cited over 50 times. Because of the delay inherent between the first appearance a papers and the possibility of its being cited, this number naturally only encompasses some of our earliest content. These early rates promise well – despite being a new journal, with emerging name recognition and without the advantage of being indexed in every relevant database, our content is nonetheless being found and read, and is influencing our field.

Two years ago when we decided to found the Journal of Cognition, our decision felt risky. Now in 2019, it is clearer than ever that open access publishing is gaining ground. Increasingly, scholars are required by their governments, institutions, and funders to ensure that their work is publicly accessible. In just a year from now, a coalition of funders will implement Plan S, which shall require their grant-holders to publish work exclusively in open-access journals where it will be immediately available, with the copyright held by the authors, not the publisher. Research funded by members of this growing coalition will need to be published in a journal that meets these key principles. This pressure toward fair open-access publishing from many of the biggest research funders in Europe will rapidly increase the demand for rigorous, high-quality fair open access outlets – like the Journal of Cognition.

Moving towards low-cost open-access need not affect the rigor of published work. Of course, both authors and funders want their work to be vetted and screened by expert reviewers to ensure its quality. As publishing shifts toward a fair open access model, scholarly societies should embrace the crucial role of disinterested gatekeeper who judges based on quality, without concern for profit. Sound editorial decisions cannot be made by someone whose main motivation is collecting article processing charges. Decisions to publish and promote research must be made by experts who can discern sound work from confounded work, and who are motivated to do that. Members of scholarly societies naturally have a stake in ensuring that the society’s journal publishes work of sound quality. Scholarly societies can ensure that their journals publish and promote only work that meets their standards. They are perfectly positioned to ensure that rigor is sustained as we shift from a profit-focused, subscription-based publishing model to an open-access model that charges a fee for processing a published manuscript.

ESCoP envisioned that a shift toward open-access publishing was coming, and Journal of Cognition is already fully compliant with Plan S. All of our content is published under a Creative Common License. All of our content is made publicly available as soon as it is produced. Our maximum article processing fee, €1150 per article, is both competitive (compare with our competitors: PlosOne charges about €1400 per paper, Frontiers charges €1600–2600) and is transparently explained on our website (https://www.journalofcognition.org/about/submissions/). A portion goes to our publisher for essential services, a portion goes to editors (who are paid a per-manuscript honorarium regardless of whether they decide to accept or reject the paper), and some of the fee is intended to defray processing charges for articles from institutions that cannot afford to pay fees. Furthermore, ESCoP members receive a discount when publishing their own work. Editorial board members, all of whom have committed to review incoming papers within their area of expertise promptly and constructively, earn a further discount for everyone at their institution. We continuously accept nominations for well-qualified editorial board members willing to take up this commitment to benefit their institution. Any profit ESCoP receives from article processing charges will be spent to promote cognitive psychology in Europe (e.g., by funding summer schools, providing small grants to defray conference travel expenses for early-career researchers, etc.).

Free, full, and fair open access is important. Europe is at the forefront of the shift toward this goal. It is up to scholarly societies like ESCoP to ensure that quality and rigor are not sacrificed in the industry-wide shift toward open access, and we are ready. In this year before Plan S is in effect, Journal of Cognition shall be working to continue developing its reputation for high-quality, fair publishing. We aim to become the top open-access choice for cognitive psychology and cognitive neuroscience. How can you help?

- If you are a cognitive scientist, especially if you are based in Europe, join ESCoP (http://escop.eu).
- Submit your own work to the Journal of Cognition. We publish review articles, reports of novel empirical findings, reports of data sets and stimulus materials, and registered reports. We are signatories of the Transparency and Openness Promotion Guidelines; you can read more about our commitment to the guidelines at journalofcognition.org.
- Consider proposing a special collection to the Journal of Cognition. The article processing charges for articles chosen for an approved special collection are defrayed by ESCoP. You may propose a collection directly to the editor by email (moreyc@cardiff.ac.uk). Proposals should include working titles of papers that will be submitted for consideration from 5–8 corresponding authors from a variety of institutions.
- Read our content regularly (https://www.journalofcognition.org/articles/). You can follow us on Twitter (@JCgntn) or Facebook or join our mailing list (http://eepurl.com/dhdAtX) for updates about new content.
- Talk to representatives at your university about fair open access, and lobby if necessary for provision for covering article processing charges at your institution.
- Register as a reviewer for Journal of Cognition by clicking on the “Become a Reviewer” button at journalofcognition.org.

Many thanks to all the authors, reviewers, and editors who contributed to the making of the inaugural volume of the Journal of Cognition. We hope that even more of you will be inspired to help us continue to make Journal of Cognition a success as we get to work on Volume 2 in 2019.
